# Hydration Layer of Only a Few Molecules Controls Lipid Mobility in Biomimetic Membranes

**DOI:** 10.1021/jacs.1c04314

**Published:** 2021-08-03

**Authors:** Madhurima Chattopadhyay, Emilia Krok, Hanna Orlikowska, Petra Schwille, Henri G. Franquelim, Lukasz Piatkowski

**Affiliations:** † Faculty of Materials Engineering and Technical Physics, Poznan University of Technology, Piotrowo 3, 60-965 Poznan, Poland; ‡ Department of Cellular and Molecular Biophysics, Max Planck Institute of Biochemistry, Am Klopferspitz 18, 82152 Martinsried, Germany

## Abstract

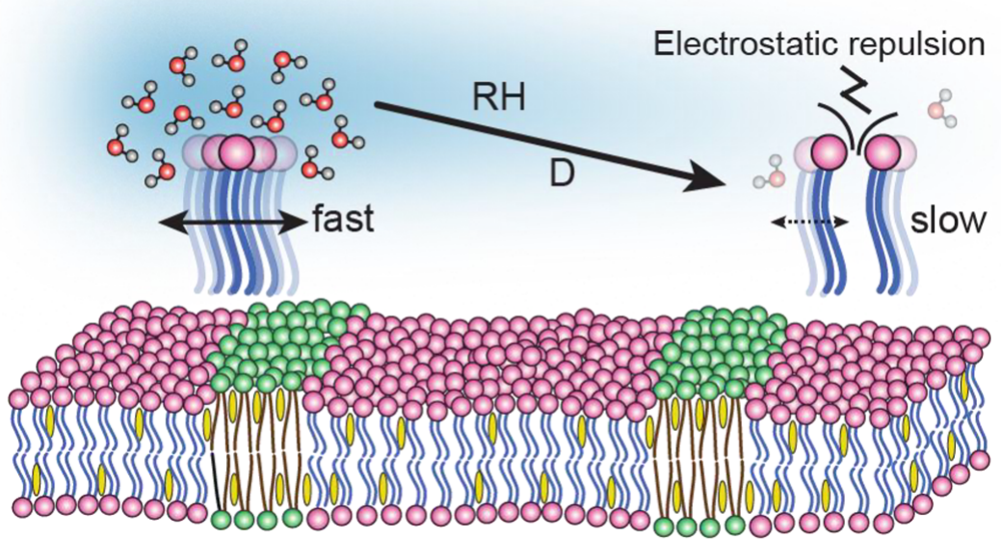

Self-assembly of biomembranes results from the intricate interactions between water and the lipids’ hydrophilic head groups. Therefore, the lipid–water interplay strongly contributes to modulating membrane architecture, lipid diffusion, and chemical activity. Here, we introduce a new method of obtaining dehydrated, phase-separated, supported lipid bilayers (SLBs) solely by controlling the decrease of their environment’s relative humidity. This facilitates the study of the structure and dynamics of SLBs over a wide range of hydration states. We show that the lipid domain structure of phase-separated SLBs is largely insensitive to the presence of the hydration layer. In stark contrast, lipid mobility is drastically affected by dehydration, showing a 6-fold decrease in lateral diffusion. At the same time, the diffusion activation energy increases approximately 2-fold for the dehydrated membrane. The obtained results, correlated with the hydration structure of a lipid molecule, revealed that about six to seven water molecules directly hydrating the phosphocholine moiety play a pivotal role in modulating lipid diffusion. These findings could provide deeper insights into the fundamental reactions where local dehydration occurs, for instance during cell–cell fusion, and help us better understand the survivability of anhydrobiotic organisms. Finally, the strong dependence of lipid mobility on the number of hydrating water molecules opens up an application potential for SLBs as very precise, nanoscale hydration sensors.

## Introduction

Biological cell membranes are dynamic barriers composed of a large variety of lipids and embedded with various proteins. Due to the complex miscellaneous molecular interactions occurring in cellular membranes, lipid model systems are particularly attractive alternatives for the controlled investigation of various physicochemical processes affecting the membrane architecture and dynamical properties. In this regard, self-assembling supported lipid bilayers (SLBs) have been well accepted as one of the most suitable model membrane systems, due to their analogous physical and structural properties to those of biomembranes and their easy preparation and handling methods.^1^ Consequently, SLBs have been exploited to investigate membrane architecture and properties such as domain formation,^2,3^ lateral diffusion or ion transport,^4,5^ and biological processes at the cellular and molecular levels, such as protein–membrane interactions,^6^ ligand–receptor interactions, cellular signaling,^7,8^ or cell adhesion.^9,10^

Various types of chemical and physical interactions determine the complex properties, architecture, and activity of the membrane. Membrane intricacy results not only from the interactions between membrane constituents, such as lipid–lipid and lipid–protein interactions, but also from the hydrophobic mismatch that arises from the interplay with water hydrating the membrane. In fact, hydrophobic mismatch is considered to be one of the key physicochemical mechanisms that regulate membrane organization and promotes nanoscopic and microscopic separation of liquid ordered (L_o_) and liquid disordered (L_d_) phases. It also determines the position and orientation of transmembrane proteins in both model and living cell membranes.^6^ The thin layer of water that directly hydrates the membrane, commonly referred to as biological water,^11,12^ has been proven to actively participate in the biological functioning of DNA.^13^ Moreover, biological water is inherently connected with the process of protein folding,^14^ aggregation,^15,16^ and stabilization of the structure even in extreme thermodynamic conditions.^17^ Numerous experiments aimed at understanding the properties of biological water, using nuclear magnetic resonance,^18^ X-ray and neutron scattering,^19,20^ infrared spectroscopy,^21^ sum frequency generation,^22,23^ and molecular dynamics simulations,^24−27^ among others, showed that water molecules form a network structure, being bound by hydrogen bonds and weak van der Waals interactions around the polar head group, the so-called clathrate hydration structure.^24^ Moreover, water molecules present in the direct or indirect hydration shell around the head group region exhibit markedly different properties from those of bulk water.^28−30^

Water is unambiguously essential for maintaining biological activities in living systems. But, in fact, nature shows various phenomena of anhydrobiosis (“life without water”) in which the cell membrane not only survives harsh dehydration but also regains full activity upon rehydration. The most common method allowing dehydration is an increased production of carbohydrates (mostly trehalose) in organisms such as tardigrades,^31−34^ nematodes,^35^ and yeasts.^36,37^ The water-replacement hypothesis states that trehalose stabilizes the head groups and enables maintaining the spacing between the fatty acyl chains.^38^ On the other hand, *Bdelloid rotifers* base their survival mechanism on the contraction of the body, which reduces the surface exposed to the environment and allows slow evaporation.^39,40^ High desiccation resistance in seeds, pollens, and anhydrobiotic plants is associated with the production of LEA (late embryogenesis abundant) proteins that are responsible for ion sequestration, protection of membranes, and renaturation of proteins that unfolded due to the lack of water.^41^

Importantly, it should be noted that local, temporary dehydration of the cell membranes also occurs continuously in our bodies during, for example, adsorption of biomacromolecules or cell–cell fusion events. A prerequisite for membrane fusion is establishing close contact between the outer leaflets of lipid bilayers such that the thin layer of water molecules is expelled (the clathrate hydration structure is disturbed) and finally overcoming the energy barrier, commonly referred to as “hydration force”, present mainly due to the repulsive forces between lipid bilayers.^42,43^

Last but not least, various studies of the electrical, mechanical, and physicochemical properties of planar lipid bilayers have revealed that these platforms have huge application potential from a technological standpoint as biosensors and biocoatings.^44,45^ These bioapplications require SLBs to be exposed to changes of external conditions such as temperature during preservation, reagent addition, and, importantly, humidity.

Hence, understanding the interplay between the hydration layers and the cell membrane is of utmost importance, both in unraveling mechanisms behind membrane organization and activity and in the frameworks of biotechnology and bioengineering. Unfortunately, the ability to investigate the intimate interactions between the membrane and the biological water has so far been hindered by the lack of appropriate experimental approaches for the preparation and study of lipid membranes in a controlled hydration state. In particular, keeping the membrane structure intact under decreased hydration conditions is challenging.^24,26^ So far, several approaches to protect the membrane from rupturing and vesiculation have been utilized: modification of lipid head groups in order to strengthen the SLB–mica attractive interactions,^46−49^ cross-linking the lipid bilayer,^50,51^ attaching polymers to the head group of lipids,^46,52^ and adding biomolecules such as proteins, disaccharides, or enzymes.^45,51,53−55^ These approaches, however, inevitably alter the intrinsic properties of the SLBs. Moreover, the exact hydration state of the membrane is unknown. Consequently, a method for preparing and stabilizing the membrane under varying, well-controlled hydration conditions without the use of additional stabilizing agents or chemical modification is needed.

Here, we present an unprecedented way to obtain phase-separated, stable SLBs with a well-controlled hydration state without interfering with membrane composition, which enables the investigation of bilayer structure and dynamics under arbitrary hydration conditions. Using a combination of fluorescence microscopy imaging and fluorescence recovery after photobleaching (FRAP) experiments, we report interesting observations of the structural and dynamical changes taking place. We show that the structure of SLBs can be preserved under dry conditions by a controlled drying process with a slow and sequential reduction in relative humidity of the membrane environment. Such an approach revealed that the lateral diffusion dynamics of the liquid disordered phase is significantly reduced with dehydration. Importantly, the membrane can undergo multiple de- and rehydration cycles always reviving its native dynamics. We also show that the diffusion activation energy for lipids in a dehydrated membrane is much higher than for fully hydrated SLBs. Finally, we provide molecular-level insights into how and which water molecules around lipids play a key role in regulating lipid dynamics in the membrane.

## Experimental Section

### Materials

1,2-Dimyristoleoyl-*sn*-glycero-3-phosphocholine (14:1 PC), egg yolk sphingomyelin (SM), 23-(dipyrrometheneboron difluoride)-24-norcholesterol (TopFluor cholesterol), and cholesterol were purchased from Avanti Polar Lipids, Alabaster, AL, USA. Monosialoganglioside (GM1) from bovine brain and 1,2-dioleoyl-*sn*-glycero-3-phosphoethanolamine labeled with Atto 633 (DOPE-Atto 633), 4-(2-hydroxyethyl)piperazine-1-ethanesulfonic acid (HEPES) sodium salt, sodium chloride (NaCl), 1,2-dioleoyl-*sn*-glycero-3-phosphoethanolamine-*N*-(7-nitro-2–1,3-benzoxadiazol-4-yl)ammonium salt (18:1 NBD PE), sodium dithionite, and chloroform (HPLC grade) were purchased from Merck KGaA, Darmstadt, Germany. Alexa Fluor 488 conjugated with cholera toxin B subunit (CTxB 488) and Alexa Fluor 594 conjugated with cholera toxin B subunit (CTxB 594) were obtained from Molecular Probes, Life Technologies, Grand Island, NY, USA. All the materials were used without further purification.

### Vesicle Preparation

The SLB was prepared by the vesicle deposition method following a formerly established protocol^56^ with suitable modification. In order to form multilamellar vesicles (MLVs), 14:1 PC, SM, and cholesterol in chloroform solution were mixed at a molar ratio 1:1:1 with the addition of 0.1 mol % of GM1 and 0.1 mol % of DOPE-Atto-633 to form a 10 mM solution of the lipids. The lipid mixture was dried under nitrogen gas, leaving a thin film of lipids deposited on the bottom of the vial, followed by desiccation under vacuum for at least 2 h. The lipids were resuspended in buffer solution (10 mM HEPES and 150 mM NaCl, pH adjusted to 7.4) and exposed to a few cycles of heating on the hot plate at 60 °C and vortexing. The lipid suspension containing MLVs was aliquoted into sterilized glass vials and diluted 10 times (final concentration of lipids 1 mM) using buffer solution. Aliquots were stored at −20 °C for further use.

### SLB Preparation

Aliquots containing MLVs of the desired composition were bath-sonicated for 10 min at maximum power to generate small unilamellar vesicles (SUVs). Freshly cleaved mica was glued to a coverslip by UV-activated glue (Norland 68), and the top layer of mica was removed right before the deposition to keep the surface properties of freshly cleaved mica intact during deposition. A half-cut Eppendorf tube was placed on the top of the coverslip and sealed with silicone. A 100 μL amount of SUV solution was deposited on top of mica followed by the addition of 2 μL of 0.1 M CaCl_2_ solution and 9 μL of 0.01 mM CTxB dissolved in buffer solution, all at room temperature. The SLB was allowed to settle for 30 s, and then 400 μL of buffer solution (10 mM HEPES and 150 mM NaCl, pH adjusted to 7.4) was added and the sample was incubated for 30 min. The bilayer was rinsed 10 times with 2 mL of buffer solution to wash out excess unfused vesicles. The Eppendorf tube reservoir was fully filled with buffer solution, closed with a glass coverslip, and sealed with silicone to prepare a fully hydrated sample containing bulk water.

### Preparation of SLBs at Different Hydration Levels

**Figure 1:**
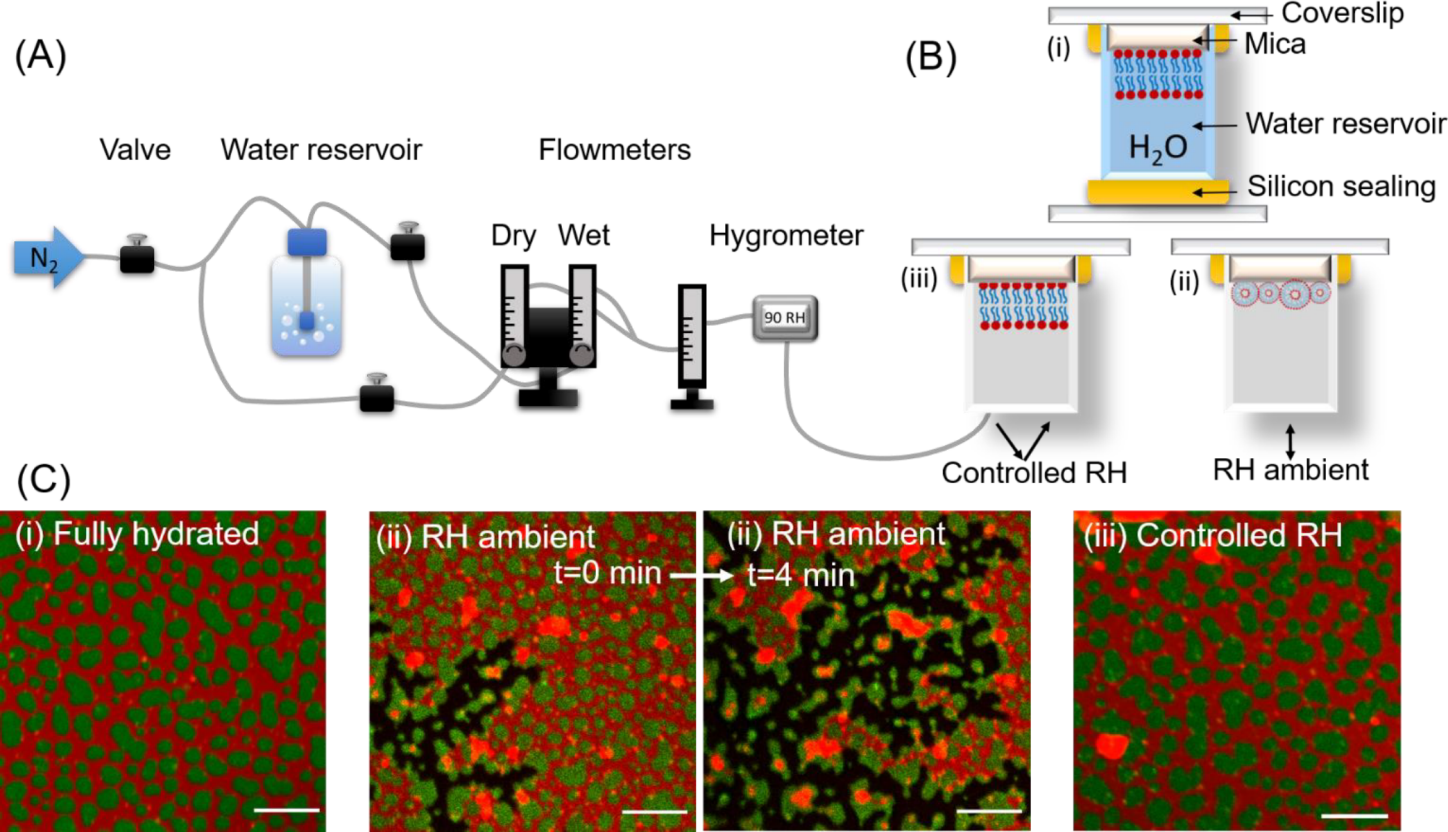
(A) Schematic representation of the home-built humidity-controlling setup. (B) Cartoon depiction of the three types of SLBs studied here: (i) fully hydrated with bulk water, (ii) exposed to the ambient humidity, where the lipid membrane curls up, forming vesicles and aggregates, (iii) exposed to atmosphere with well-controlled humidity, which at complete dehydration resembles the fully hydrated membrane. (C) Fluorescence images of the representative SLBs exhibiting phase separation into L_d_ (labeled with Atto-633-DOPE, shown in red) and L_o_ (labeled with CTxB-Alexa488, shown in green) domains in different hydration conditions indicated in panel B. The two middle panels show progressive rupturing and delamination of the SLB abruptly exposed to ambient RH. Upon dehydration the lipid membrane detaches from the solid support, leaving areas of bare mica (black). The curled-up membrane forms big clusters composed of both phases, which are visible as orange and yellow aggregates. Scale bar corresponds to 10 μm.

In our work, two distinct methods were implemented for drying the SLBs. (a) The bulk water was pipetted out and the sample was left open to dry and equilibrate to atmospheric humidity (∼30% RH) at room temperature, and (b) after removal of bulk water by micropipet the sample was equilibrated in an atmosphere of different relative humidity (RH%). An atmosphere of different relative humidity was created inside the open half-cut Eppendorf tube by purging nitrogen gas of a specific relative humidity using a home-built control unit (see Figure 1A). The setup consisted of three flow meters, three manual valves, a reservoir with water, and an electronic hygrometer with 0–95% RH range and 1% precision. The relative humidity of nitrogen gas was adjusted and maintained by mixing a suitable amount of wet (saturated with water vapor, 90% RH) N_2_ and dry (2–3% RH) N_2_ gas. The electronic hygrometer was used to monitor the final relative humidity and temperature of the N_2_ gas being purged toward the sample. To study the SLB structure and dynamics at different relative humidity, the silicone seal of the sample was cut, water was pipetted out completely, and purging of wet nitrogen gas of 90% RH was started immediately toward the SLB. The RH was decreased (and subsequently increased) in steps of ∼20% at a rate of 2–3% RH per minute. Next, the SLB was equilibrated at a given RH for about 10 min before FRAP measurements were performed. The relative humidity of wet nitrogen gas was decreased gradually from 90% (62 × 10^19^ water molecules/min) to approximately 70% (48 × 10^19^ water molecules/min), 50% (34 × 10^19^ water molecules/min), and 30% (20 × 10^19^ water molecules/min), and finally dry nitrogen (around 2–3% RH) was purged to the SLB. Similarly, rehydration of the dried SLB was done by purging wet nitrogen gas with increasing relative humidity and finally resealing the half-cut Eppendorf tube filled with water.

### Fluorescence Microscopy and FRAP

Laser-scanning confocal imaging and FRAP experiments were performed on SLBs using an upright Zeiss LSM 710 (Carl Zeiss, Jena, Germany) microscope with a 40× 1.3 NA oil immersion objective. Lasers of wavelengths 488 and 633 nm were used for excitation of Alexa Fluor 488 and Atto-633-DOPE, respectively. In the case of 3-fold labeling with TopFluor cholesterol, CTxB-Alexa Fluor-594, and Atto-633-DOPE, lasers of 488, 543, and 633 nm were applied accordingly. Laser power was adjusted during imaging to avoid excessive photobleaching of the sample. A small circular spot of 10 μm diameter was bleached, and the area of the bleached spots was kept constant for all FRAP experiments. Diffusion coefficients were calculated by fitting the fluorescence recovery curve considering free Brownian lateral diffusion of lipid molecules in the membrane using the modified Soumpasis formula:^57^*F*(*t*) = *b* + *a* × *f*(*t*), where *a* is the amplitude of the recovery function, *b* is the remaining fluorescence after bleaching, and *f*(*t*) is the Soumpasis function. Fitting was done for data normalized with respect to the reference intensity signal of the whole image excluding the bleached spot. A complete dehydration and rehydration cycle was performed for three samples, and FRAP experiments were performed in at least five different spots at a particular relative humidity for each sample. Diffusion coefficients were averaged over a range of RH at which particular traces were measured.

### Temperature Dependence Experiments

Variation of *D*(*T*) was examined for two hydrated and two dehydrated samples in the temperature range 25 ± 1 to 45 ± 1 °C. A resistive tape was attached to the sample reservoir tube for heating, and a thermocouple was placed inside the reservoir tube for continuous monitoring of sample temperature. Activation energies for the two samples were calculated using the Arrhenius equation: ln *D* = ln *A* – *E*_a_/*RT* where *D* is the diffusion coefficient, *A* is the pre-exponential factor (assumed to be temperature independent in this range), *R* is the universal gas constant, and *T* is the temperature in Kelvin scale. For Arrhenius plots, weighted linear regression of ln *D* values was presented. The confidence bounds generated by the fitting of FRAP traces were considered as error bars for *D* and their reciprocals to be the weights.

## Results

### Structure

In this study, the changes in the membrane structure at different hydration conditions have been examined by fluorescence imaging. In the experiments we considered three levels of membrane hydration, described in detail in the Experimental Section and schematically depicted in Figure 1B: (i) fully hydrated SLB, where the membrane is submerged in bulk water, (ii) SLB for which most of the bulk water was pipetted out and the sample was left open to equilibrate to room humidity (∼30% RH), and (iii) SLB for which bulk water was removed to the highest extent and the sample was immediately exposed to a N_2_ atmosphere with ∼90% RH.

Fully hydrated SLBs, marked as (i) in Figure 1B,C, exhibit homogeneously distributed domains of liquid-ordered (L_o_) phase with an average area of 1.77 ± 0.29 μm^2^. The domain size, distribution, and shape are typical of L_d_/L_o_ phase-separated SLBs prepared in such conditions, in full agreement with previous reports.^58^ SLBs are far from static; over time they slowly merge with each other to form bigger domains. Over the course of ∼24 h the average domain size increases by up to 40%.

The SLB (marked as (ii) in Figure 1B,C) for which most of the bulk water was removed and the surface was exposed to open air of low RH (∼30%) initially exhibits an identical structure to the fully hydrated sample. Compared to fully hydrated SLBs, here we observed an increase in vesicle-like aggregated structures, mainly composed of L_d_ phase residing on the surface of the SLB. However, as spontaneous drying proceeds, the remnant bulk water layer shrinks, causing the drop-like macroscopic water layer wavefront to pass over the membrane surface. The local changes of surface tension induce delamination of the membrane from the mica support (the two middle panels in Figure 1C). Intriguingly, in most cases, the L_d_ phase detaches from mica first, while L_o_ domains remain attached to mica (extended time series is shown in Figure S1 and in Movie M1). Shortly after, over the course of a few minutes, also L_o_ domains shrink and form curled-up vesicle-like structures mixed with the L_d_ phase lipids. Delamination of the membrane ceases as soon as the residual bulk water is evaporated. However, it should be noted that even when the process of dehydration is conducted in a rapid manner, in several areas confined by mica terraces the SLB structure remains unperturbed (Figure S2). This mechanism of membrane preservation in the presence of mica terraces as mechanical supports is explained in more details in the Discussion section.

**Figure 2:**
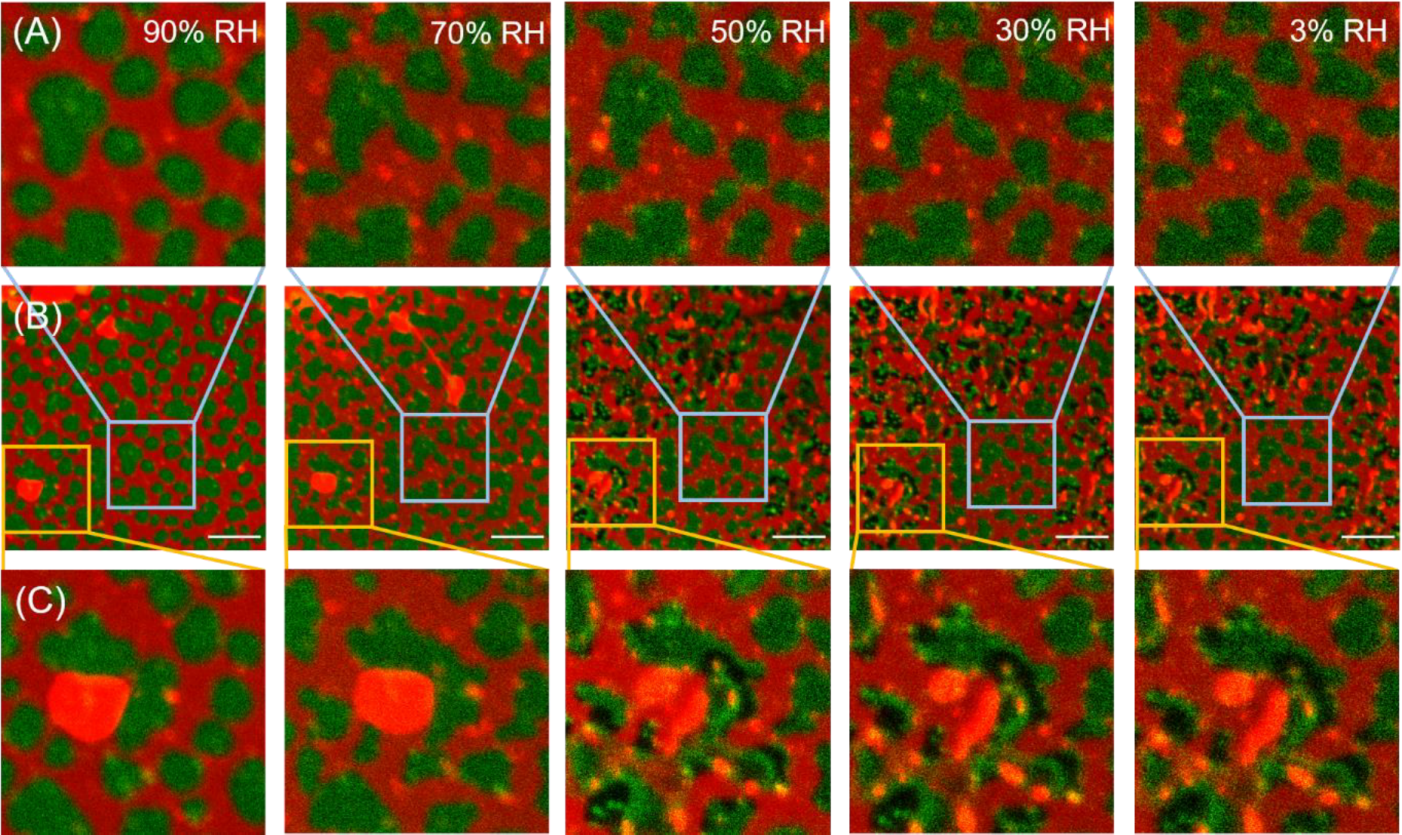
Consecutive fluorescence images of the same area of SLB exposed to 90%, 70%, 50%, 30%, and 3% RH. Top row (A) and bottom row (C) show the zoomed-in region indicated by the blue and yellow rectangles in images in the middle row (B), respectively. Equilibration time for each hydration condition and between consecutive images was ∼30 min. The L_d_ phase is labeled with Atto-633-DOPE (red), and the L_o_ phase is labeled with CTxB-Alexa488 (green). Scale bar corresponds to 10 μm.

Markedly different behavior was observed when the SLB was exposed to a N_2_ atmosphere with a high RH of ∼90%, directly after bulk water removal. The SLB kept under a continuous flow of a N_2_ atmosphere with high RH (denoted as (iii) in Figure 1B,C) qualitatively closely resembles a fully hydrated SLB. Minor delamination was observed solely on the perimeter of the sample, close to the mica edges. This curling up of the membrane occurs during the time required to remove bulk water and expose the membrane to an atmosphere of high RH. These events are likely responsible for the increased number of vesicles and aggregates at the top of the membrane (Figure 2A–C). The aggregates that appear due to bulk water removal are initially mobile and float while the residual water evaporates. Once the sample equilibrates with an atmosphere of high RH (70–80% RH), the aggregates become stagnant. No change in the structure or quality of the SLB kept in such conditions was observed over the course of a few hours. No significant change in the quality of the membrane structure was noticed, although the perimeter of domains became increasingly jagged with further, gradual decrease of the RH down to about 50%.

At around 50% relative humidity, the appearance of seemingly hole-like dark spots within L_o_ domains (labeled with CTxB-Alexa 488) is observed at several locations on confocal microscopy images. In the range of 50% through 30% to nearly 0% RH, the membrane structure does not change significantly except for the appearance of dark spots in L_o_ domains in a few more locations. Noticeably, the formation of these hole-like dark spots is limited to a few areas, while an unperturbed and continuous phase-separated membrane structure can be observed over the prevalent sample area even at a relative humidity close to 0%. Evidently, by means of a slow, well-controlled, and gradual (∼2–3% RH/min; for details see the Experimental Section) decrease of membrane hydration, an air-stable membrane can be formed without the addition of external stabilizing agents. Additional confocal images of the sample as a function of hydration are shown in Supporting Information Figure S3. On lowering the hydration below 50% RH, the big aggregates (ranging from 5 to 25 μm^2^), located at the top of the membrane, break into smaller ones.

It should be noted that the membrane equilibrated at different hydration states is stable for up to a few hours. Intriguingly, the process of dehydration is fully reversible; that is, the dehydrated membrane can be rehydrated back to the state compliant with high RH and further to full hydration by addition of bulk water (Figure S4). Upon rehydration, the darker spots within L_o_ domains become homogeneously bright again and the domains regain their former (rounder) shapes at around 70–85% RH. Images of SLBs at different RH during rehydration are shown in Figure S3.

### Dynamics

Next, we examined whether the hydration state of the membrane affects the mobility of the lipids by performing FRAP experiments on membranes equilibrated at different hydration conditions. The mobility of lipids constituting the membrane depends on the composition of the SLB.^59^ The measured single-component fully hydrated membrane of 14:1 PC shows a higher diffusion coefficient (2.93 ± 0.44 μm^2^/s) than the L_d_ phase of our, phase-separated SLB (∼1.7 μm^2^/s), which is consistent with the previous reports.^60^

**Figure 3:**
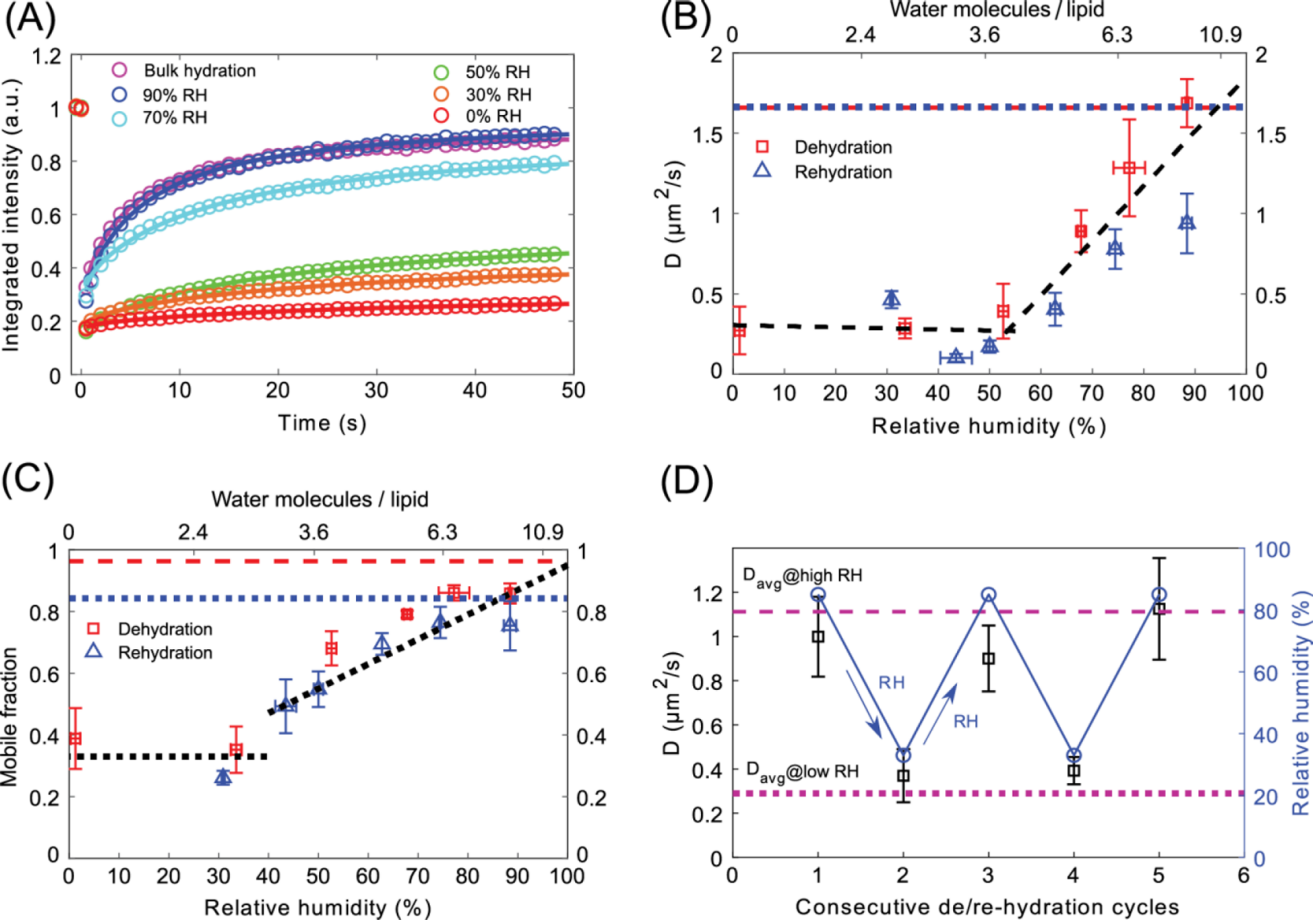
(A) FRAP traces of fully hydrated SLB and SLB equilibrated to 90%, 70%, 50%, 30%, and 0% relative humidity. (B) Diffusion coefficient for the L_d_ phase for SLBs at different relative humidity during dehydration (red squares) and rehydration (blue triangles). The data points correspond to the diffusion coefficient averaged from at least 5 FRAP traces from each of the 3 samples at a particular RH. The two black dashed lines are separate linear regressions of the data points at >55% RH and at <55% RH. The red dashed and blue dotted lines correspond to diffusion coefficient for a fully (bulk) hydrated SLB (averaged over three different samples) before dehydration and after rehydration, respectively. (C) Mobile fractions extracted from the fits of the modified Soumpasis formula (see Experimental Section) to the FRAP traces during dehydration (red squares) and rehydration (blue triangles). The black dotted lines are guides to the eye highlighting the data changes similar to those in panel B. The red dashed and blue dotted lines correspond to mobile fractions for a fully hydrated SLB (averaged over three different samples) before dehydration and after rehydration, respectively. (D) Diffusion coefficient (black squares) averaged over 5–7 FRAP traces at each hydration level during consecutive dehydration and rehydration (87% ↔ 33% RH) cycles (blue circles). Purple dashed and dotted lines correspond to the average diffusion coefficient for all the measured FRAP traces for SLBs kept at high (85–90% RH) and low (30–35% RH) relative humidity, respectively.

FRAP traces obtained for the phase-separated SLBs in different hydration states are shown in Figure 3A. Evidently, with lowering hydration of the membrane, the mobility of the L_d_ phase decreases significantly. At a fully hydrated condition, i.e., before removal of bulk buffer solution, the L_d_ phase lipids showed the highest mobility of 1.66 ± 0.22 μm^2^/s. After the withdrawal of bulk water and being equilibrated to ∼90% RH, the mobility remained unaltered. With a further decrease in hydration level, the diffusion coefficient (*D*) of lipids has been observed to decrease prominently (Figure 3B). The average *D* decreases over 6 times during dehydration from 1.69 ± 0.29 μm^2^/s for 87 ± 2% RH to 0.27 ± 0.29 μm^2^/s at 3 ± 2% RH. A steady decrease in the mobility of lipids is observed from full hydration to around 50% RH. Below 50% RH, the mobility of L_d_ lipids remains almost constant. The fluorescence recovery for fully hydrated membranes and membranes equilibrated with high RH% (∼90%) reaches 93 ± 3% of the initial fluorescence intensity. At a relative humidity less than 85% the fluorescence does not recover up to the initial intensity, and in the case of RH lower than 50%, fluorescence recovery is significantly lower and amounts to less than 50% of the initial fluorescence intensity. The extracted mobile fraction, defined as the amplitude of the fitted recovery function normalized to the total bleach depth (), as a function of (de)hydration state of the membrane, is shown in Figure 3C.

Interestingly, during rehydration of the SLB, by increasing the relative humidity level gradually from 0% to 90%, the mobility of lipids increased accordingly and was strongly correlated with the diffusion coefficient observed during dehydration of the membrane (Figure 3B).

The extracted mobile fraction during the rehydration process also closely resembles that observed during dehydration for each specific hydration state. Upon a full dehydration/rehydration cycle, both the average *D* value and mobile fraction reach their initial values. Taking all the data into account, we observed two regimes. In the range of 50–90% RH, *D* exhibits significant changes with hydration. On the other hand, below 50% RH, *D* is nearly independent of the hydration of the membrane. The linear regressions performed on the data points in these two ranges show a clear turnover point at about 50% RH. A similar trend is observed for the mobile fraction: a significant decrease above 50% RH and little dependence in the hydration range below 50% RH.

Consecutive cycles of drying and rehydrating the SLB in the range of 87% to 33% RH were performed three times on the same sample while at each hydration state recording FRAP traces from a minimum of six spots. The sample was equilibrated for 10 min at a particular RH%. Remarkably, once bulk water is completely removed, the membrane exhibits very good stability in terms of structure and full reversibility of its dynamics. Keeping the membrane in such conditions allows strong modulation of the mobility by a factor of nearly 4: ∼0.3 μm^2^/s vs ∼1.2 μm^2^/s (see Figure 3D).

In accordance with previous reports the diffusion rates of L_o_ and L_d_ phases are significantly different: 1.66 ± 0.22 μm^2^/s for L_d_ and 0.1 ± 0.01 μm^2^/s for L_o_. While qualitatively it appears that the diffusion coefficient decreases for the L_o_ phase when lowering a membrane’s hydration, it is very difficult to quantify this change in a reliable manner for two reasons: (a) the diffusion coefficient is already very low at full hydration, as it corresponds to the diffusion of the GM1-CTxB-AlexaFluor complex, where one CTxB molecule binds to 1–5 units of GM1,^61^ leading to the diffusion of few lipids at the same time, and (b) the signal-to-noise ratio of the signal is quite low due to the much lower (4–8 times) fluorescence quantum efficiency of the CTxB label at low hydration conditions (see Figure S8). However, to still address the mobility of the L_o_ phase under different hydration conditions, we used an alternative fluorescent label (TopFluor cholesterol), which participates in both L_o_ and L_d_ phases with a roughly 80:20 ratio, respectively.^62^ To this end, we prepared membranes with only the L_o_ phase, composed of cholesterol and SM at a molar ratio of 1:1. The obtained FRAP traces and extracted diffusion coefficients are presented in Figure S5. We observed that the mobility of the L_o_ phase decreases with the lowering of the membrane hydration, following the same trend as for the L_d_ phase. It can be concluded that although the absolute values of the diffusion coefficient for the more dynamic L_d_ phase and the less mobile L_o_ phase are different, the response of both phases to the hydration changes is similar.

## Discussion

### Structure

The multicomponent SLBs composed of 14:1 PC, SM, and cholesterol exhibit substantial structural changes with abrupt dehydration, but remain largely intact at lower hydration conditions when subjected to a well-controlled, gradual decrease in hydration level.

After bulk dehydration, the membrane is covered with a remnant, thin layer of water that desorbs over time. Exposing the membrane to the ambient RH causes the residual water to evaporate rapidly, causing fast shrinking of the water layer and inducing delamination and curling up of the membrane followed by lipid aggregation (see Figure 1C, Figure S1, and Movie M1). This is due to the domination of the air–water interfacial force over the attractive forces between the mica substrate and the proximal leaflet of the SLB.^45^ Detachment and curling up of the L_d_ phase prior to the L_o_ phase during drying can be explained by differences in mechanical properties of the two phases. The L_o_ phase is stiffer (higher bending modulus and area expansion modulus) than the L_d_ phase,^63^ which results in lower steric forces and stronger interaction with the substrate. The observed stronger interaction of the L_o_ phase with mica than the L_d_ phase is consistent with the stronger adhesive interactions observed for DSPC gel phase domains, reported in previous research.^64^ It should be noted that the probability of survival of SLBs during rapid drying is increased by the presence of intrinsic defects of the support, such as mica terraces and/or cleaving defects (see Figure S2). The defects obstruct the drying water, decreasing the local water–air tension and protecting the membrane from delamination. This observation is in accordance with the previous report on preparation of an air-stable membrane by generating an obstacle network made of peripheral enzyme phospholipase A_2_ as physical confinement, where the presence of defects affects the local surface tension and stops the water–air from propagation, leaving the membrane intact.^45^

In contrast, for the SLB exposed and equilibrated to high relative humidity (∼90%) the overall membrane structure remains largely unaffected, except for the deposition of a few aggregates on top of the bilayer (see Figure 2). Upon decreasing the relative humidity further in the range of 90–55%, we observed no significant changes to the structure of the membrane: the SLB still exhibits homogeneously distributed L_o_ domains in an L_d_ matrix. With decreasing hydration, however, the perimeter of the L_o_ domains becomes increasingly jagged (see Figure 2A). The ragged outlines of the L_o_ domains are mostly evident in the AFM topography image acquired on a membrane equilibrated to 30% RH (see Figure S6). AFM studies of fully hydrated SLBs of analogous composition showed round L_o_ domains with a smooth perimeter.^65^ Moreover, the thickness difference between the L_d_ and L_o_ phases for a dehydrated SLB is nearly 3 times lower (∼0.6 nm, see Figure S6) compared to the thickness mismatch for a fully hydrated SLB with the same composition (∼1.56 ± 0.13 nm).^65^ Clearly, lowering the hydration of the membrane leads to a decrease in the hydrophobic mismatch between the L_d_ and L_o_ phases and consequently of the line tension.

At lower hydration conditions (<50% RH), dark spots in some of the L_o_ domains appear (see Figure 2C), where fluorescence of the labeled GM1-CTxB complex is not detected. At the same time, parts of these L_o_ domains exhibit locally higher fluorescence intensity. Detailed analysis of the fluorescence images reveals the nature of the dark spots within the L_o_ domains. The shape (outline) of the domains before the appearance of the dark spots (RH > 50%), with the dark spots present (RH < 50%), and after the disappearance of the dark spots (upon rehydration) remains the same (see Figure S7). If the dark spots were due to the formation of holes within the membrane, one would expect that upon rehydration the shape would randomly change; that is, the holes would be filled randomly by the L_d_ and/or L_o_ phase. Instead, we observe that the L_o_ domains maintain their original shape and regain a fluorescence distribution as before the dehydration.

Next, we analyzed the fluorescence intensity of selected L_o_ domains containing the dark spots as a function of hydration. The total integrated fluorescence intensity of an L_o_ domain before, during, and after filing the dark spots remains the same and is only affected by the overall photobleaching of the dye (see Figure S8). Thus, the dark spots do not result from the local bleaching of the CTxB label, but rather from the local redistribution/aggregation of the GM1-CTxB complexes.

More detailed insights and the proof for the aggregation of the CTxB within L_o_ domains comes from fluorescence images with the 3-fold labeling. We kept the labeling of the L_d_ and L_o_ phases (DOPE-Atto and GM1-CTxB, respectively), but we added fluorescently labeled cholesterol (TopFluor), which should partition in both L_d_ and L_o_ phases (see Figure S9). As expected, for domains that exhibit a homogeneous distribution of CTxB within the L_o_ domain, we observe homogeneous colocalization of CTxB and labeled cholesterol within the L_o_ phase. For domains that exhibit aggregation of CTxB, we still observe the homogeneous distribution of the labeled cholesterol. This unambiguously proves that the local appearance of dark areas within the L_o_ phases is solely related to CTxB aggregation and not to structural changes of the membrane. While the exact reason behind the CTxB aggregation remains elusive, it should be noted that it is mainly observed where aggregates of other membrane constituents on top of the membrane are present. We also note that at about 50% RH, aggregates on top of the membrane break into smaller pieces, likely taking up the energetically more favorable structure at the anhydrous conditions. Intriguingly, when increasing the hydration state of the membrane, the homogeneous fluorescence signal within the L_o_ domains is recovered, indicating that the distribution of the GM1-CTxB complexes becomes homogeneous (Figure S3).

It is evident that the dehydration process itself, when carried out in a controlled manner, does not affect the structure of the SLB. Such preserved membrane structure-wise remains insensitive to dehydration and rehydration cycles. This conclusion is consistent with the recent molecular dynamics simulations study, which for a strongly dehydrated lipid bilayer reported the presence of four bridging water molecules per lipid (discussed in detail later). These strongly H-bonded water molecules at the interior of the membrane (bound to a carbonyl and/or phosphate group) contribute strongly to the structural and mechanical integrity of the membrane.^66^

### Dynamics

With a decrease in hydration level, the mobility of L_d_ lipids decreases. As evident from Figure 3B, we find that the diffusion coefficient decreases between the fully hydrated sample and the fully dehydrated sample by over a factor of 6 (from 1.69 to 0.27 μm^2^/s), which confirms a major role of water in lipid dynamics.

Upon removal of bulk water when the SLB is equilibrated to a humid environment (90% RH), the diffusion coefficient remains unchanged and the fluorescence intensity recovers to a similar extent after photobleaching, as in the case of fully hydrated SLB (Figure 3C). This implies that the fluidity of L_d_ lipids remains unhindered in the absence of bulk water and that water molecules present per lipid at 90% RH are sufficient for the lipids to retain their native (read in full hydration) mobility. This is understandable, as at high RH membrane constituents can coordinate as many water molecules as it is energetically most favorable, likely completely filling their direct hydration shell. The biggest changes to the diffusion coefficient are observed with lowering the RH down to about 50%. Further lowering of RH brings little change to the diffusion coefficient.

So far we assumed that the measured lipid mobility reflects the entire bilayer, that is both the upper and lower leaflet. However, the literature is inconsistent as to whether the lipids in the upper and lower leaflet of a bilayer exhibit similar diffusional dynamics. Hetzer et al. showed that for bilayers on silica beads the diffusion coefficient of lipids in the upper monolayer is roughly 2 times higher than for lipids in the lower monolayer.^67^ On the other hand, studies by Zhang and Granick^68^ showed that regardless of whether the DLPC bilayers were deposited on quartz or on a polymer cushion, *D* was the same for the outer and inner leaflet within the experimental uncertainty. To address this issue, we redesigned the experiment and used DOPE coupled with NBD dye, which undergoes irreversible fluorescence quenching upon addition of sodium dithionite,^69^ allowing the detection of lipids from only the lower leaflet. Addition of sodium dithionite to the fully hydrated membrane indeed leads to a 2-fold decrease in mean fluorescence intensity of the membrane, indicating that the upper leaflet is quenched and the fluorescence signal only comes from the lower leaflet (Figure S10A). The diffusion coefficient (Figure S10B) and extracted mobile fractions (Figure S10C) are nearly identical before and after quenching, revealing that for the used, fully hydrated membrane diffusional dynamics of the upper and lower leaflet are very much alike. The diffusion coefficient for the half-quenched bilayer shows a strong decrease with dehydration of the membrane (see Figure S11B). The measured roughly 5-fold decrease in *D* is very similar to the ∼6-fold decrease of *D* with dehydration in the case of FRAP acquired for both leaflets, giving a clear indication that the two leaflets respond very similarly to the dehydration. Hence in what follows we assume similar mobility and hydration properties of lipids in the upper and lower leaflet. However, we note here that the fluorescence signal intensity during dehydration process shows a significant increase (Figure S11A). This originates from an increase in the fluorescence quantum yield of NBD dye at lower hydration conditions.^70,71^

**Figure 4:**
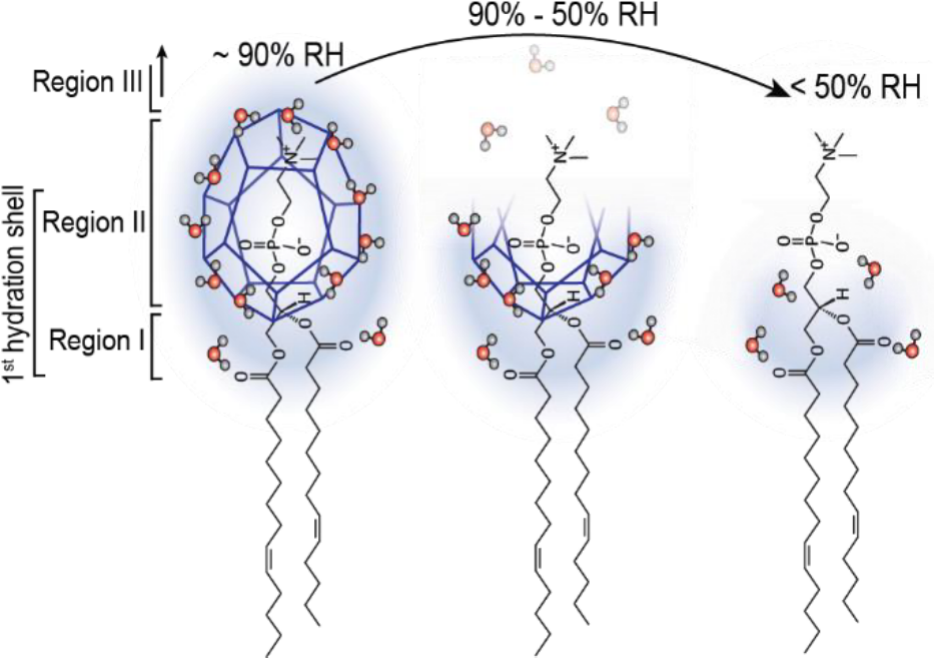
Schematic description of stepwise detachment of water molecules from the three regions of a 14:1 PC lipid during a controlled dehydration process.

In order to understand the changes in the diffusion coefficient under varying hydration conditions, we need to consider the hydration structure of individual lipid molecules. Phosphatidylcholine (PC) molecules are zwitterionic lipids containing a positively charged choline ((CH_3_)_3_N^+^CH_2_CH_2_OH) moiety and a negatively charged phosphate (PO_4_^3–^) group. Three distinct regions have been identified (Figure 4) within PC, where water molecules are bound either by weak van der Waals interactions or by H-bonds.^72^ Region I corresponds to the interior water molecules, buried deeper in the membrane and forming H-bonds with carbonyl oxygens of the glycerol region. Region II refers to water molecules forming a cage-like (clathrate) structure around the whole phosphocholine group. Finally, the consecutive hydration shells/bulk water around the head group belong to region III.^25^ Molecular dynamics (MD) simulations of a PC bilayer revealed that there are 10–12 water molecules in the first hydration shell, and among these, up to three water molecules remain tightly attached to lipids (in the glycerol and/or phosphate region), even after drastic drying.^24^ Another MD simulation by Gierula et al. showed that nonesterified oxygen atoms of the phosphate group form about four H-bonds and the two carbonyl oxygen atoms form about one H-bond each;^26^ thus approximately six H-bonded water molecules per PC are present in the first hydration shell (regions I and II). The choline group cannot form a H-bond with water molecules; instead it remains surrounded by a clathrate hydrate containing ∼6.4 water molecules,^26^ held via weak electrostatic and van der Waals interactions.

The experimental studies using X-ray diffraction (XRD) and infrared spectroscopy also showed that upon bulk water dehydration of stacked lipid bilayers and equilibration of the system at ∼95% RH there are around 11 to 12 water molecules per lipid, confirming quantitatively the structure of the first solvation shell around the lipid group.^21,73^ Both theoretical and experimental studies are thus consistent as to the number of water molecules (∼12) per PC lipid in the first solvation shell. The same experimental works determined that a further decrease in RH (95% → 75% → 50% → 25%) of the environment of bilayer stacks leads to a lowering of the hydration of lipids to approximately 10.9, 6.3, 3.6, and 2.4 water molecules per lipid (averaged from the two experimental works), respectively. Naturally, the desorption of water molecules should occur according to their H-bonding energies. Previous studies reported that H-bonds between the water molecule and the phosphate group are stronger than water–carbonyl group H-bonds, while both of these H-bonds are stronger than interwater molecule H-bonds.^72^ Therefore, water molecules loosely bound with weak van der Waals interactions, as well as bound to other water molecules, will detach first, followed by a detachment of water molecules bound through the strongest H-bonds to phosphate and/or carbonyl moieties. This is in accordance with the molecular dynamics simulation results, which showed that, upon dehydration, water–water hydrogen bonds break, while the lipid–water bridging hydrogen bonds persist.^66,74^

Supplementing our experimental observations with the considerations above, a clear picture of the interplay between the water and the lipid membrane emerges (Figure 4). Upon withdrawal of bulk water and equilibration of the SLB with high RH, outer solvation shells are removed and only the first, direct solvation shell containing around 12 water molecules per lipid remains. Under these conditions, the diffusion coefficient of the L_d_ phase remains unaffected. Clearly, the water molecules beyond the first hydration shell are not involved in the mobility regulation of the lipids in SLBs. When decreasing the RH down to ∼50%, a sharp and continuous drop in the mobility of L_d_ phase lipids occurs. In this regime, each lipid loses six to seven water molecules. This implies that the clathrate structure breaks apart because at 50% RH only about four water molecules are left, which is insufficient to form a stable cage around the phosphate moiety. In the regime below 50% RH the lipid mobility hardly changes. Apparently, the remnant two to four water molecules tightly attached to phosphate and, in particular, carbonyl oxygen atoms do not affect lipid mobility. Interestingly, both molecular dynamics simulations and experimental works revealed that the diffusional and orientational mobility of the strongly bound water molecules is diminished at low hydration states.^21,74^ These findings strongly correlate with our current observation of the very low mobility of lipids at low hydrations. It is evident that out of the water molecules within the first solvation shell, those forming the clathrate structure are mostly involved in facilitating the lateral diffusion of the lipids in SLBs.

After establishing which water molecules contribute to the regulation of the lateral mobility of lipids, the question arises of why and how these water molecules affect the mobility of lipids. For each diffusion step, a lipid molecule needs to possess energy higher than the activation energy of diffusion (*E*_a_) and to have sufficient free volume in the vicinity.^75^ Free volume in our SLBs could decrease if small perforations (or nanoholes) were formed in the bilayer in a dehydrated condition. However, fluorescence images and AFM topography images (Figure S6), revealing a flawless and uniform L_d_ phase in dehydrated SLBs, nullify this scenario. Therefore, the activation energy factor must dominate here.

**Figure 5:**
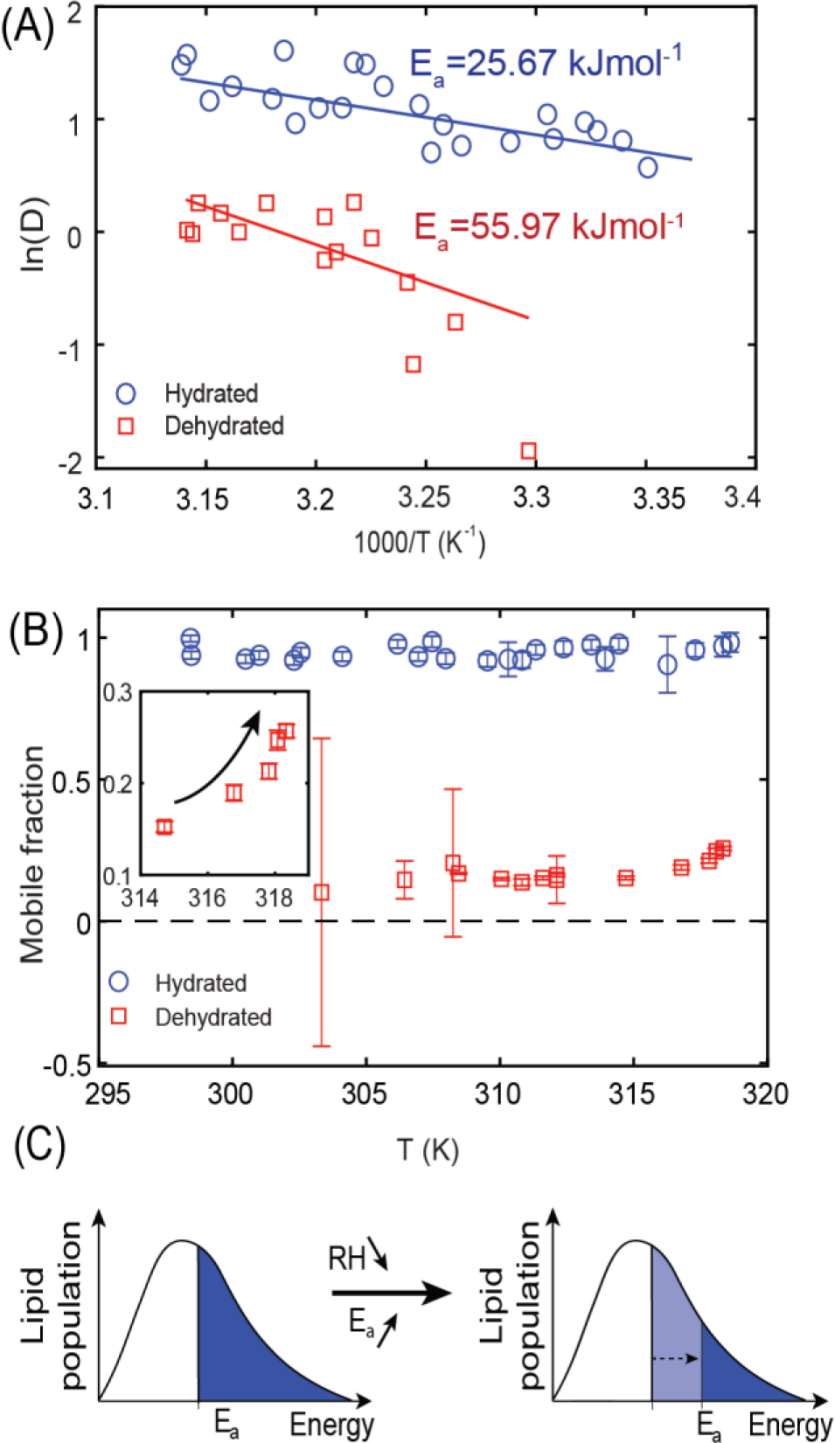
(A) Arrhenius plots for one representative fully hydrated (blue circles) and dehydrated (red squares) SLB. (B) Temperature dependence of the mobile fraction extracted from the data shown in panel A. (C) Schematic representation of the relation between the diffusion activation energy and the lipid mobile fraction in the dehydration experiment.

Water molecules forming the clathrate screen the repulsive Coulombic interactions between adjacent lipid head groups.^24,76^ Consequently, in the absence of this shielding water cage, the repulsive interactions between adjacent head groups become more prominent, increasing the activation energy for diffusion. In other words, for dehydrated SLB, a lower population of lipids possesses sufficient energy to overcome the diffusion activation energy barrier. Consequently, the probability for a lipid molecule to overcome the activation energy barrier at a particular time decreases, leading to an overall decrease in diffusion coefficient and mobile fraction. We confirmed this by measuring the activation energy for diffusion for fully hydrated and dehydrated (∼30% RH) SLBs. Figure 5A depicts representative Arrhenius plots for hydrated and dehydrated SLBs. *E*_a_ for fully hydrated bilayers averaged over four data sets (two SLBs, increasing and decreasing temperature for each SLB) amounts to 23 ± 4 kJ mol^–1^, which is consistent with the previous reports.^77^*E*_a_ for a dehydrated membrane, averaged over four data sets, is approximately twice as high and amounts to 47 ± 17 kJ mol^–1^. An increase of *E*_a_ for a dehydrated lipid monolayer has been qualitatively predicted earlier based on theoretical considerations.^78^ The higher standard deviation of *E*_a_ for the dehydrated sample results from higher uncertainty in fitting the very slow fluorescence recovery in the FRAP data. Evidently, decreasing hydration of the SLB leads to a noticeable increase in activation energy for the L_d_ phase.

Importantly, the significant increase in *E*_a_ for diffusion with dehydration explains the observed decrease in mobile fraction during dehydration. As *E*_a_ increases, the population of molecules having enough energy to overcome the barrier at a particular time decreases (Figure 5C), which is reflected in the slower recovery of FRAP traces and lower mobile fraction. In this case, an increase in mobile fraction should be observed with increasing the temperature, as more energy is delivered to the lipids. For a fully hydrated sample, the mobile fraction is already greater than ∼95% and there is very little or no room for it to increase further. In the case of the dehydrated SLB, the mobile fraction indeed tends to increase at higher temperatures (Figure 5B).

Altogether, the observed slowing down of the diffusion with an increase in diffusion activation energy suggests that the SLB quasi-gellifies (stiffens) upon dehydration, in particular upon the removal of the clathrate water molecules. This is in agreement with previous studies, which indeed suggested that dehydration leads to an increase in the main phase transition temperature of lipids,^79,80^ indicating fluid-to-gel-like transition at lower hydration conditions.

Finally, we note that with rehydration the former mobility of lipids is restored. The variation of diffusion coefficient with hydration state for a few dehydration and rehydration cycles demonstrates that the mobility of lipids strongly depends on the availability of the water molecules per lipid, and the diffusion coefficient is instantaneously responsive toward water content. It is also evident that, for the intact membrane that survived the dehydration process, losing or gaining mobility of lipids due to change in hydration is completely reversible and repeatable.

## Conclusions

We successfully prepared desiccation-tolerant, phase-separated lipid bilayers without mechanical or chemical alterations. While a rapid reduction in water content causes irreversible damage to the SLB structure, a gradual and controlled dehydration process allows the preparation of stable SLBs even in the complete absence of water. Dried SLBs can be brought back to full hydration without affecting their integrity and reused as functional membranes after rehydration. Thus, storage and handling of such desiccation-tolerant SLBs become much easier for bioengineering applications such as biocoatings.

We carefully addressed the structural and dynamical properties of SLBs across a wide spectrum of hydration states. While structurally, SLBs showed little sensitivity to the hydration state of the SLB, we observed a 6-fold decrease in lateral diffusion coefficient for the lipids forming an L_d_ phase with lowering hydration of the SLB. Importantly, we correlated the observed changes of the diffusion coefficient with the lipid hydration structure and established that these are six to seven water molecules hydrating the phosphocholine head group and forming a cage-like structure that acts as a lubricant for the diffusion and modulates the lateral mobility of disordered phase in the SLBs. We demonstrate that the observed slowing down of the diffusion is directly related to an increase in activation energy for diffusion at lowered hydration conditions. Together with the unpredicted overall structural stability, these findings point toward quasi-gellification of the SLB with lowering its hydration. Intriguingly, the native dynamics of the fully hydrated SLB is recovered with rehydration. Consequently, the dried SLB with unperturbed membrane structure and dramatically reduced mobility can be considered as a less active form of the membrane, which can be compared with the dormant stage of organisms exhibiting anhydrobiosis. Local “anhydrobiosis” occurs also in our organisms during for instance cell–cell interactions or during binding of large biomacromolecules, when the water molecules are expelled from the interaction site. It is thus conceivable that the observed slowing down in SLB dynamics also occurs locally and leads to stiffening and stabilization of the membrane, potentially stabilizing transient molecular interactions.

Our studies on the interplay between the membrane and its hydration open up a range of exciting experiments that could certainly provide new molecular-level insights into effects such as hydrophobic mismatch, line tension, or the properties of the interphase boundaries of the membrane structural complexes.

Finally, a clear relation between the diffusion coefficient and the number of water molecules hydrating the membrane could be utilized for biosensing applications to monitor the local hydration state of biomimetic systems. This idea further gains in impact when the hydration sensing is done on a single-molecule/probe level using, for example, fluorescence correlation spectroscopy or single-particle tracking techniques. Our findings thus hold a huge application potential from both biological and technological viewpoints.

## Supporting Information Available

Supplementary Figures S1–S11 (PDF)
Supplementary Movie 1 (MP4)
