# Supplementary material for: Hydration Layer of Only a Few Molecules Controls Lipid Mobility in Biomimetic Membranes
Source: J Am Chem Soc. 2021 Aug 3;143(36):14551–62. doi: 10.1021/jacs.1c04314 (PMC8447254; doi:10.1021/jacs.1c04314)
Supplement: Supplementary file 1 — ja1c04314_si_001.pdf [file ja1c04314_si_001.pdf]

Supplementary Information for

Hydration layer of only few molecules controls lipid mobility in biomimetic membranes

Madhurima Chattopadhyay,[1]* Emilia Krok,[1] Hanna Orlikowska,[1] Petra Schwille,[2] Henri G. Franquelim,[2] and Lukasz Piatkowski[1]*

[1]*Faculty of Materials Engineering and Technical Physics, Poznan University of Technology, Piotrowo 3, 60-965 Poznań, Poland*
[2]*Department of Cellular and Molecular Biophysics, Max Planck Institute of Biochemistry, Am Klopferspitz 18, 82152 Martinsried, Germany*

Table of contents:

Supplementary figures S1 – S11.

Figure S1

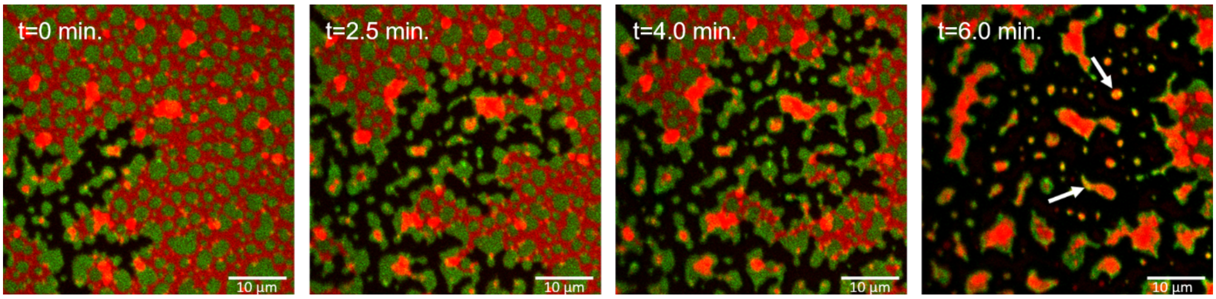

Fig. S1. Consecutive fluorescence images of the same area of SLB exposed abruptly to ambient RH. Desorption of water causes shrinking of the remnant water layer and induces delamination of the lipids from the mica substrate (black). During drying, the water layer wavefront passes over the surface and causes detachment of the L_d phase (red). L_o phase (green), which has a stronger affinity to the mica substrate, stays unperturbed for a longer time. With time both phases pill off the solid support. Dried SLB (rightmost image) contains aggregates composed of both phases (yellow spots, marked with white arrows).

Figure S2

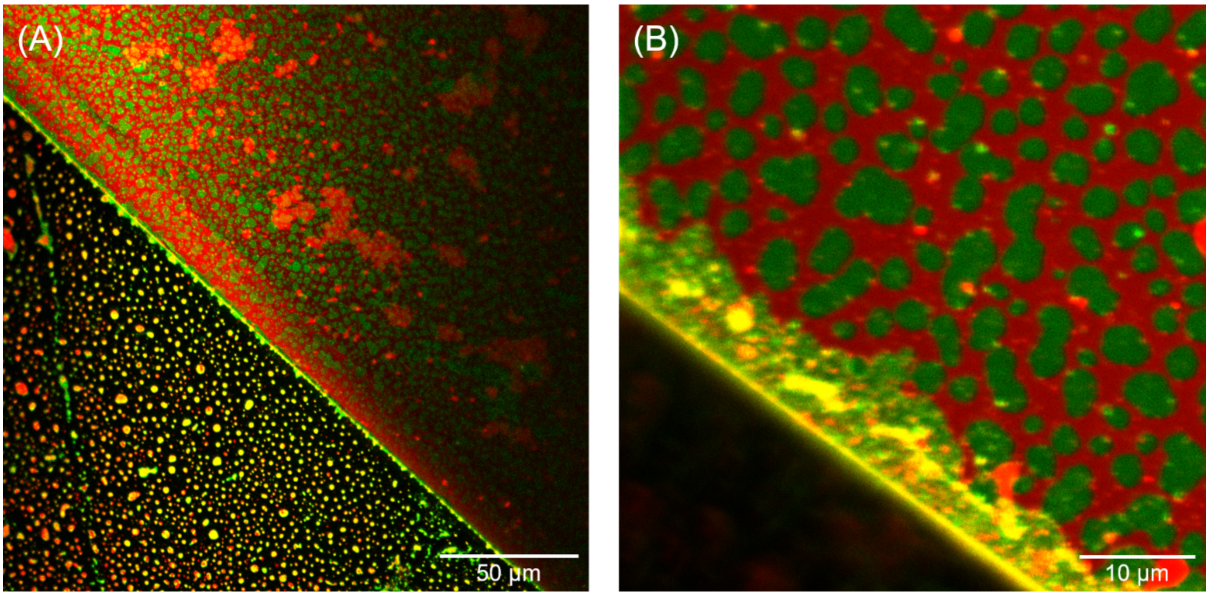

Fig. S2. The presence of defects of the solid support (mica cleaving-induced defects) can influence membrane stability upon rapid dehydration. Mica terraces stop the drying waterfront and act as an obstruction preventing the membrane from delamination. (A) Membrane curls up in the bottom-left part of the image, forming vesicles and aggregates (orange and yellow), and remains unperturbed in the upper-right part section of the image, (B) Close-up image of the border between the two regions. During dehydration, the dragged lipids were deposited by the moving waterfront along with the defect. In case of rapid dehydration, the membrane curls up in the absence of the mica terraces, which act as mechanical obstruction. In stark contrast, during controlled dehydration membrane remains unperturbed irrespective of the presence of the mica terrace as shown in Figure S3 and S4.

Figure S3

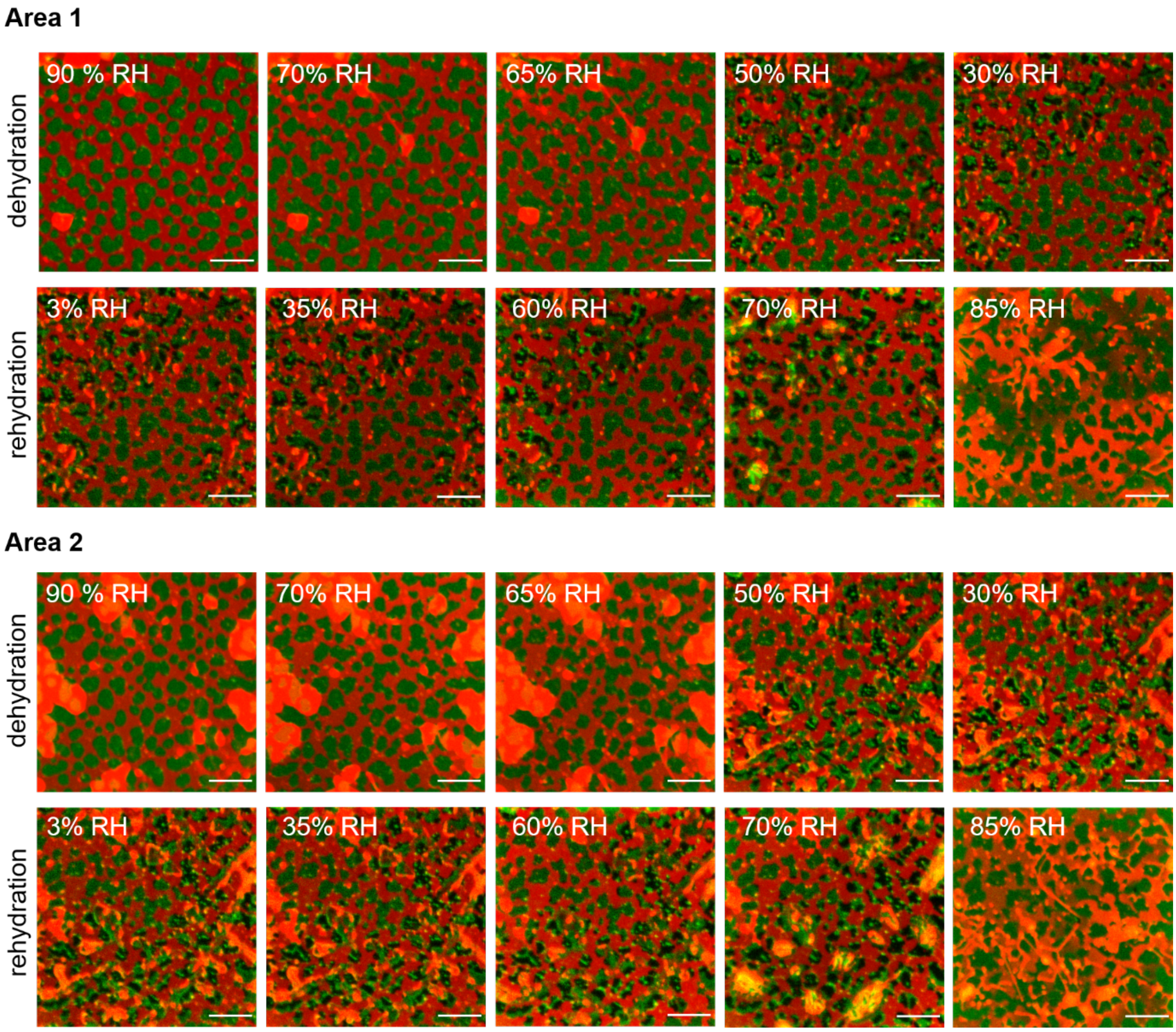

Fig. S3. Consecutive fluorescence images of two different areas (area 1 and area 2) of SLB during dehydration (top row) and rehydration (bottom row). At the humidity of around 50% RH and lower, in some areas of the membrane, local aggregation of the GM1-CTxB occurs, which is visible as the formation of darker and brighter spots within the L_o domains. At about 50% RH aggregates (orange and yellow) accumulated on top of the membrane break into smaller ones. During rehydration, L_o domains regain homogeneous CTxB distribution (and hence homogeneous fluorescence signal) at about 85% RH. The scale bar is 10 µm.

Figure S4

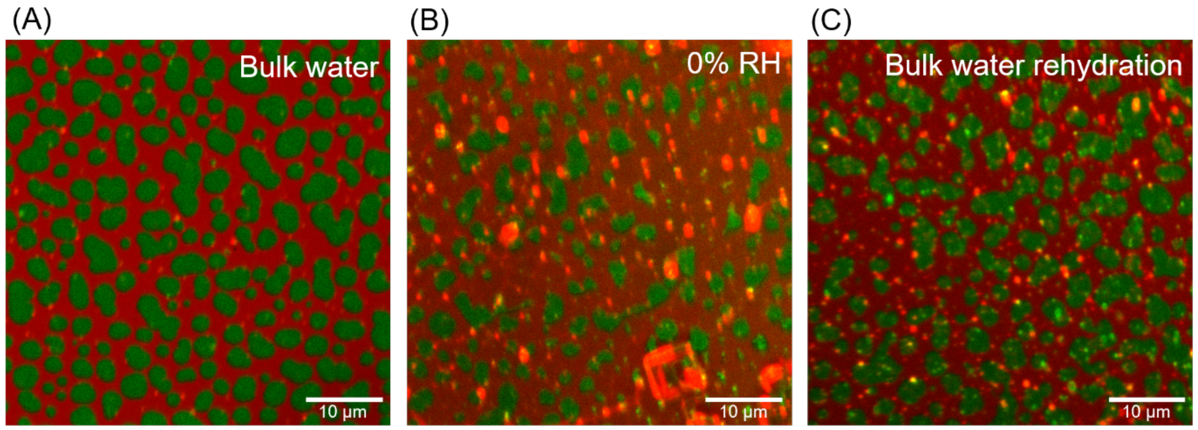

Fig. S4. Confocal images of the same sample recorded at different hydration conditions: (A) right after preparation, full hydration with bulk water, (B) slowly dehydrated by decreasing the humidity level and kept at 0% RH, (C) slowly rehydrated and refilled with bulk water. Membrane structure remains unaffected after a complete cycle of de(re)hydration.

Figure S5

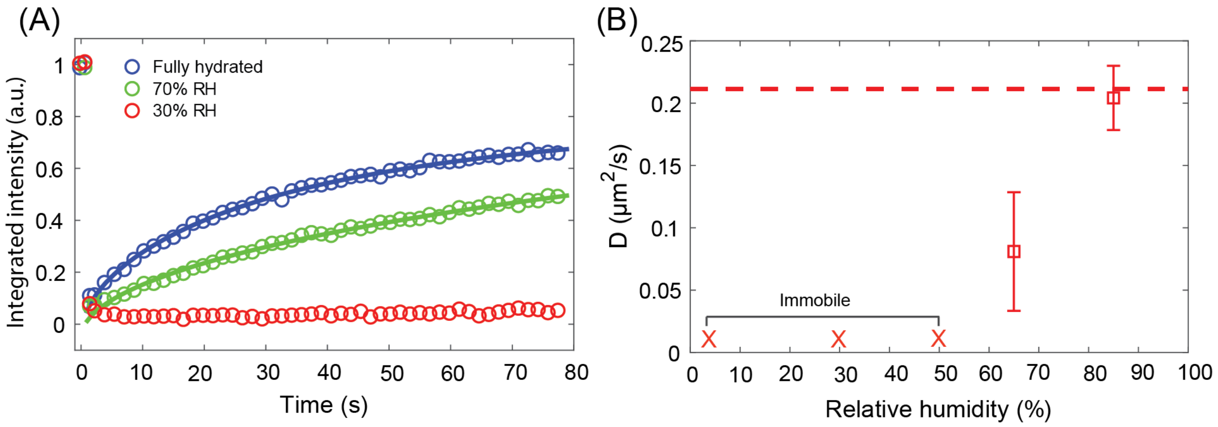

Fig. S5. FRAP traces and diffusion coefficient of the L_o phase labeled with TopFluor cholesterol as a function of membrane hydration. The single component membrane was composed of only SM and cholesterol with the same ratio as in the phase separated membranes (1:1 molar ratio). (A) FRAP traces of the fully hydrated SLB and SLB equilibrated at 70% and 30% relative humidity. (B) The diffusion coefficient for the L_o phase for SLB at different relative humidity during dehydration. The red dashed line corresponds to the diffusion coefficient of the L_o phase at full hydration conditions (bulk water hydration). FRAP traces for lipid membrane at 50%, 30% and 3% RH could not be reliably fitted due to the lack of fluorescence recovery. Values of diffusion coefficient for these hydration conditions are indicated here as immobile (red X). Notably, the changes of the diffusion coefficient as a function of hydration exhibit the same trend as those presented for the L_d phase (see Fig. 3B), indicating that both phases respond to the dehydration process in a similar manner.

Figure S6

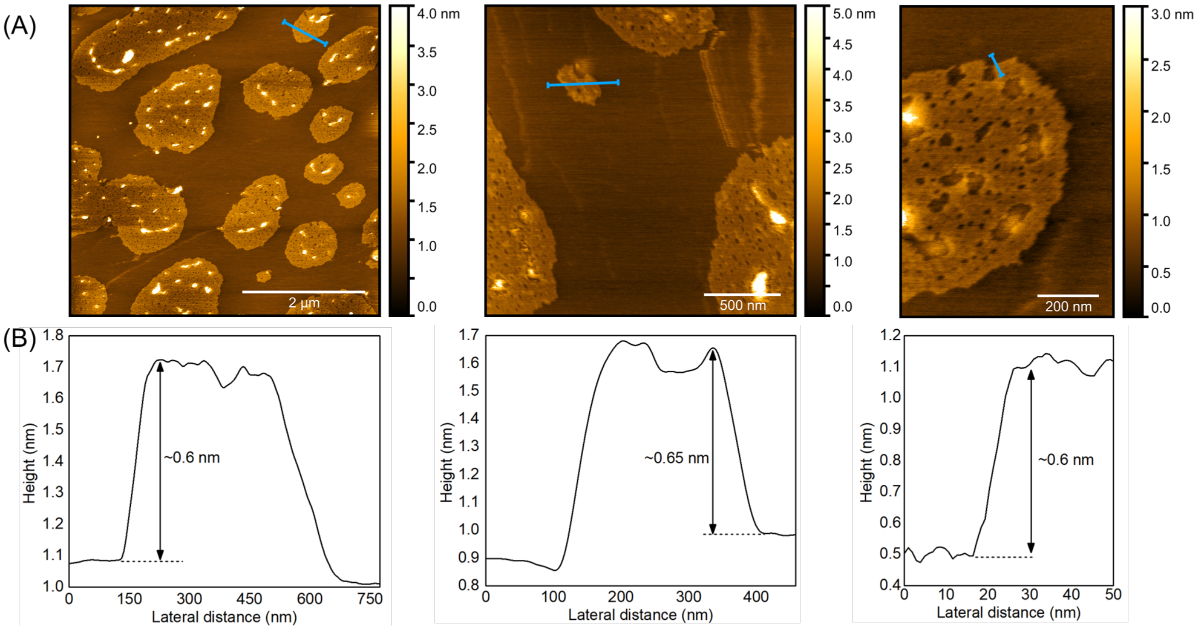

Fig. S6. AFM images of dehydrated SLB. (A) Topography scans confirming intact membrane with continuous L_d phase at dehydrated condition. Presence of nanoscopic depressions with a depth of ~0.5-0.7 nm is noticed in L_o phase domains. Given the same height difference as between the L_o and L_d phases, these depressions correspond to L_d nanodomains trapped in the L_o phase. A similar effect was observed for ceramide-rich membranes, where due to lower line tension of the L_o phase, L_d nanodomains participated in the L_o domains[1]. Elevated features with the height of ~6 nm (from the average surface height of L_o domains) correspond to the GM1-CTxB complexes. Scale bars are 2 µm, 0.5 µm and 0.2 µm for the left, middle and rightmost image, respectively. (B) Cross-sections profiles highlighting the height difference between the L_d and L_o phase (marked by blue lines in panel A).

Figure S7

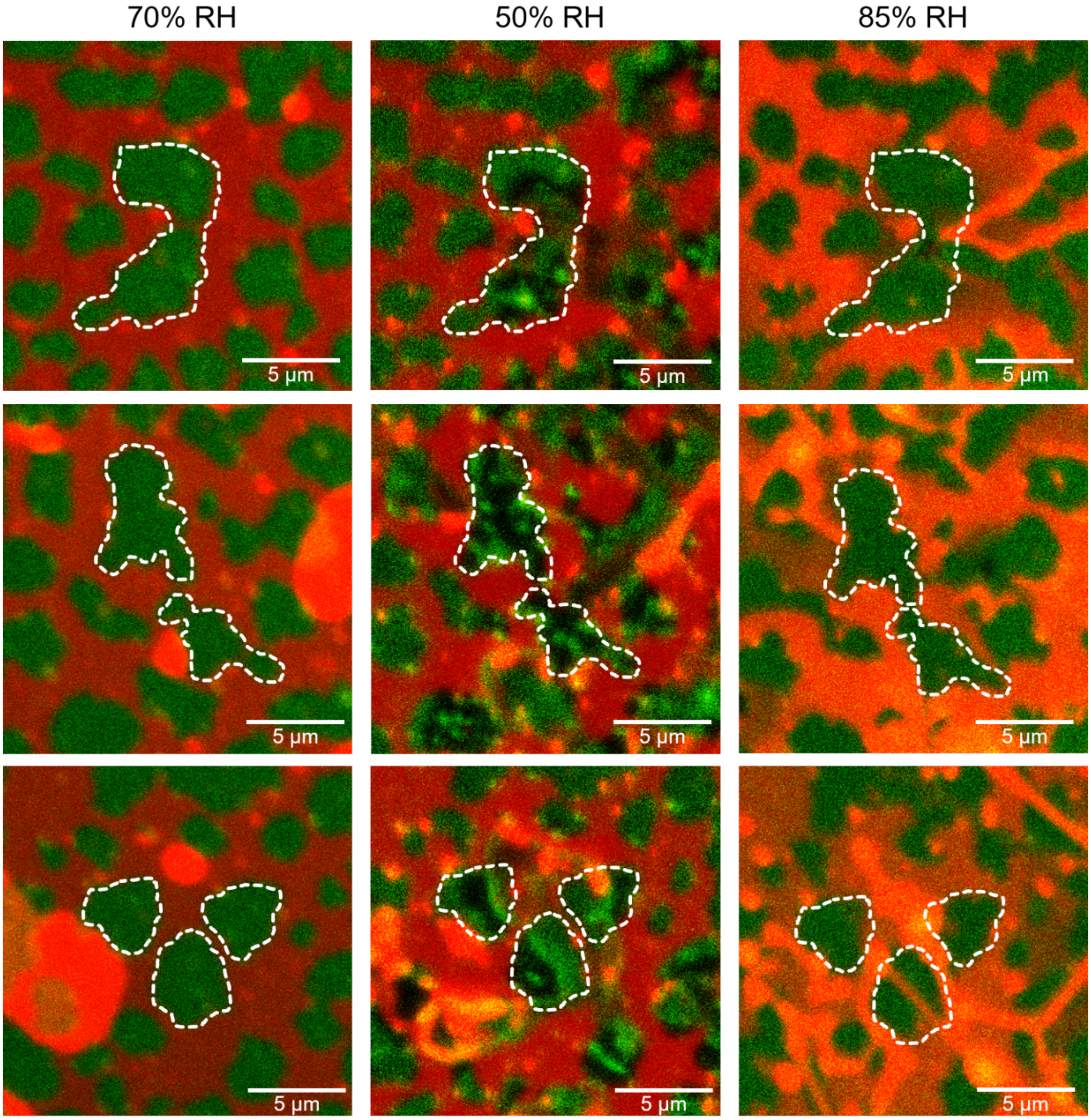

Fig. S7. SLB consisting of L_d (red) and L_o (green) phase exposed to the relative humidity of 70% and 50% (during dehydration) and 85% (during rehydration). The white, dashed lines mark the outlines of the domains. The shape and area of the domains during the dehydration and rehydration processes remain largely the same. The GM1-CTxB complexes aggregate at around 50% RH but upon rehydration redistribute again evenly within the whole domain.

Figure S8

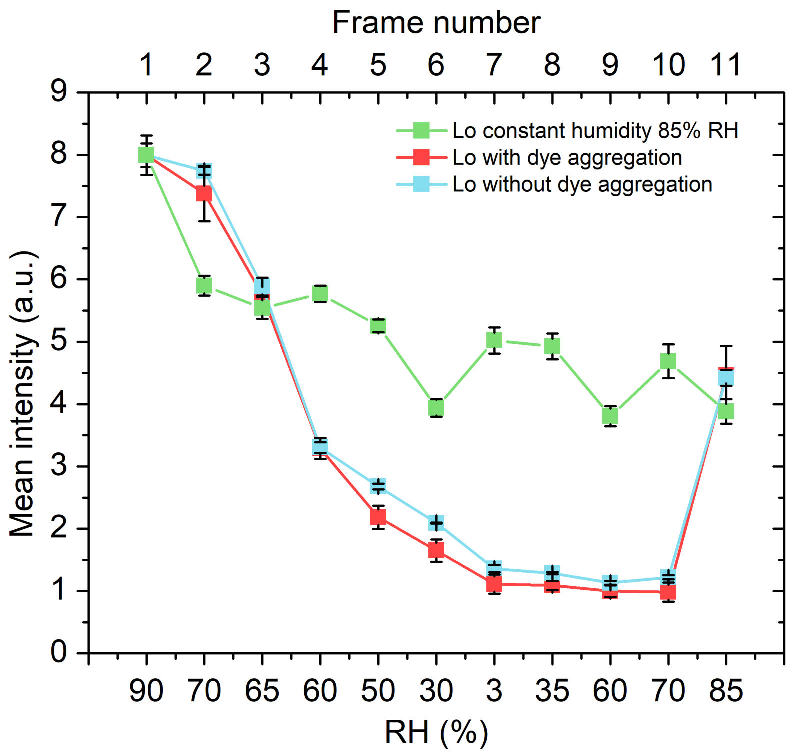

Fig. S8. Changes in the mean intensity of the L_o domains with de(re)hydration. Green trace reflects the native fluorescence bleaching of the Alexa Fluor 488 dye caused by the consecutive imaging of the SLB exposed to the constant humidity of 85% RH. The mean intensity is plotted as a function of frame number (top x-scale). Red trace shows the changes in the fluorescence intensity of the domain showing aggregation of the GM1-CTxB complexes during dehydration and rehydration. Light blue trace represents the changes in the domain intensity for a domain that did not show any aggregation of the GM1-CTxB complex. The overall fluorescence intensity for the domains with and without the aggregation changes in the same manner but it shows a more rapid intensity decrease than for the sample kept in constant humidity. Upon rehydration, at 75-85% RH, the fluorescence of the domain is restored. The final mean intensity of the fluorescence after taking 11 images at constant humidity is the same as after the whole de-/rehydration cycle. Evidently, the strong decrease in fluorescence of the Alexa Fluor 488 dye is due to the lower quantum efficiency of the dye at lower hydration and not solely due to photobleaching of the dye. Lipid domains were analyzed using ImageJ software by measuring the mean intensity from the chosen L_o domains (three showing GM1-CTxB aggregation and three with homogeneous GM1-CTxB distribution). Intensities were normalized with respect to their initial value.

Figure S9

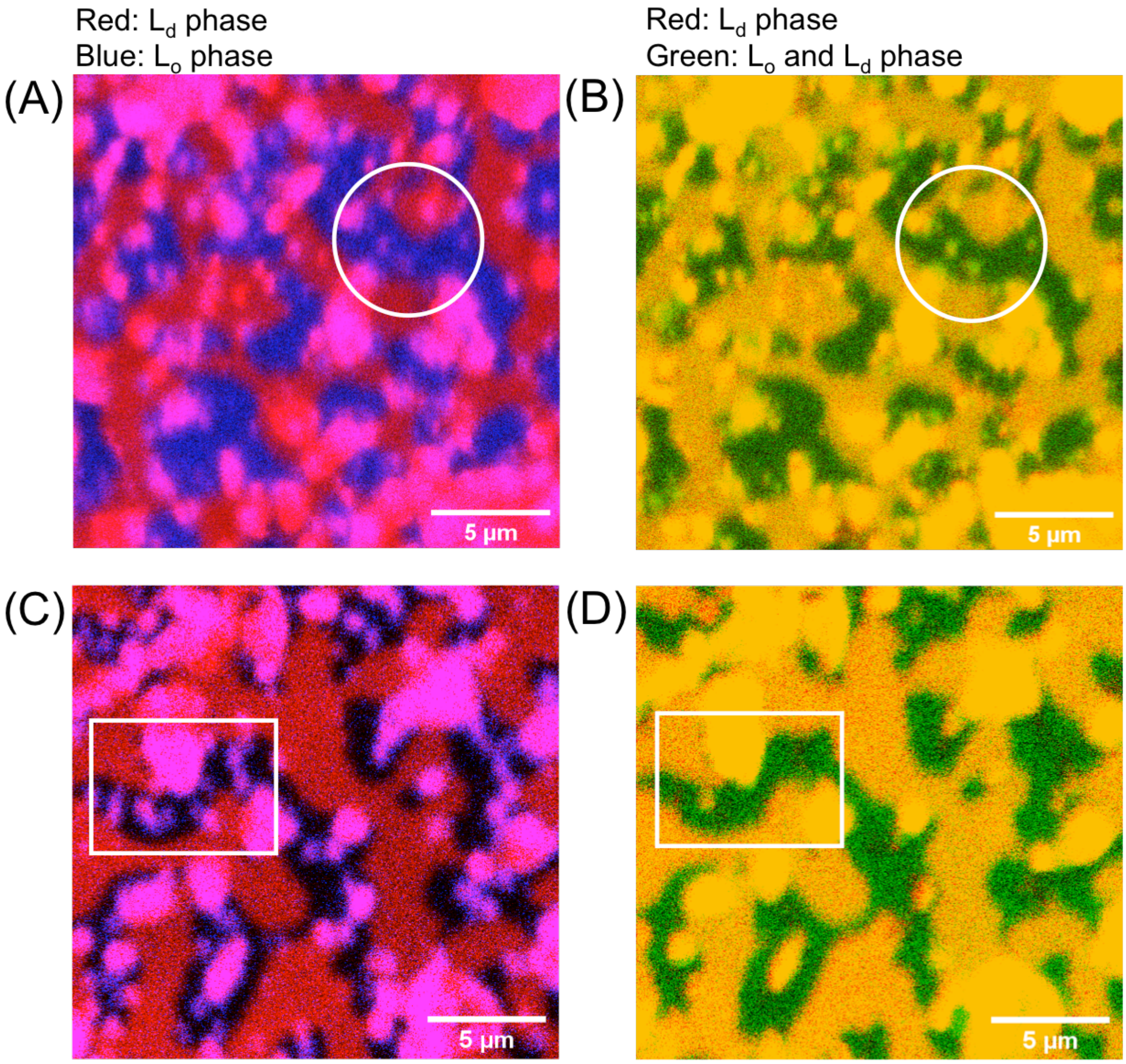

Fig. S9 Confocal images of two areas of SLB equilibrated at low relative humidity (~30% RH), where L_o domains show homogeneous (top row, A-B) and inhomogeneous (bottom row, C-D) distribution of the GM1-CTxB. (A, C) L_d phase is labeled with Atto-633-DOPE (red) and L_o phase is labeled with CTxB-Alexa594 (blue). (B, D) L_d phase is labeled with Atto-633-DOPE (red), while cholesterol, which partitions in both L_d and L_o phase is labeled with the TopFluor dye (green). L_d phase appear yellow in panels B and D, as it overlaps homogeneously with the green signal assigned to the cholesterol label. It is clear that, while in some areas GM1-CTxB tends to aggregate leaving dark (no fluorescence) spots within the L_o domains (panel C, white rectangle), the structure of the L_o domains themselves remains intact. This is evident from the unperturbed, homogeneous distribution of the cholesterol-TopFluor (panels B, D).

Figure S10

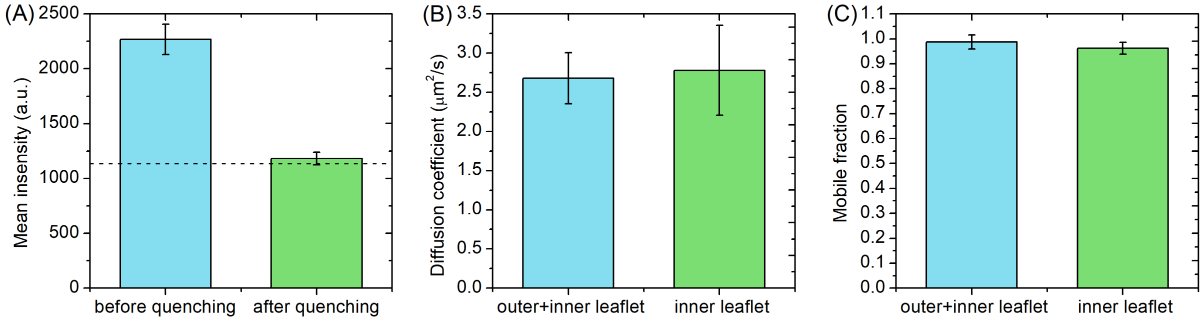

Fig. S10 Analysis of the mobility of outer and inner leaflet at fully hydrated condition. (A) Mean fluorescence intensity averaged over 10 confocal images for the bilayer (blue bar, indicated here as "before quenching"), and for the inner leaflet only (green bar, described as "after quenching"), obtained after the dithionite quenching of the NBD-labeled lipids. The dashed, black line indicates 50% level of the mean fluorescence intensity before quenching. The mean intensity shows a 2-fold decrease (green bar) upon addition of sodium dithionite. (B) Diffusion coefficient of L_d phase for both outer (upper) and inner leaflet (lower/support facing) and for inner leaflet only, averaged over 6 FRAP traces. (C) Mobile fraction of L_d phase for both leaflets (blue) and for inner leaflet only (green) - values were averaged over 6 FRAP traces. These observations confirm that both outer and inner leaflets behave in similar way for our system at fully hydrated condition.

Figure S11

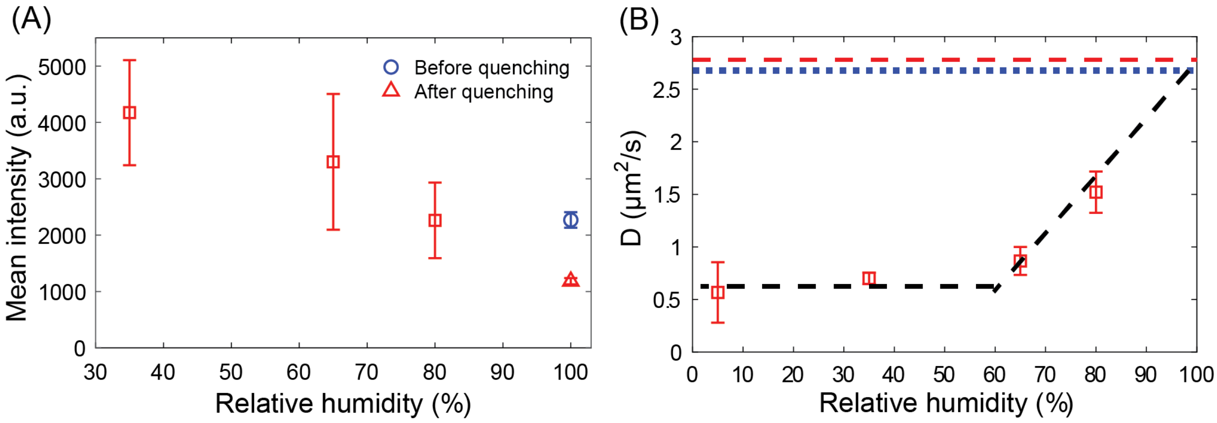

Fig. S11 Mean intensity of SLB with quenched upper leaflet and mobility of the lower leaflet as a function of hydration. (A) The mean intensity of the SLB decreases by a factor of two upon dithionite addition (as decribed in S10). Upon dehydration mean intensity of SLB with quenched upper leaflet increases as the quantum yield of NBD dye increases at dehydrated conditions[2,3]. (B) The diffusion coefficient of the inner leaflet as a function of hydration condition. D was averaged over 6 FRAP traces at each hydration level. The blue dashed line shows the average diffusion coefficient for the bilayer at fully hydrated condition. The red dashed line corresponds to the average diffusion coefficient at full hydration for the inner leaflet only. Change in diffusion coefficient for the lower leaflet during dehydration follows the same trend as for full bilayer.

References:

(1) Kol, M.; Williams, B.; Toombs-Ruane, H.; Franquelim, H. G.; Korneev, S.; Schroeer, C.; Schwille, P.; Trauner, D.; Holthuis, J. C.; Frank, J. A. Optical Manipulation of Sphingolipid Biosynthesis Using Photoswitchable Ceramides. *Elife* **2019**, *8*.

(2) Mazères, S.; Schram, V.; Tocanne, J. F.; Lopez, A. 7-Nitrobenz-2-Oxa-1,3-Diazole-4-Yl-Labeled Phospholipids in Lipid Membranes: Differences in Fluorescence Behavior. *Biophys. J.* **1996**, *71*, 327–335.

(3) Fery-Forgues, S.; Fayet, J. P.; Lopez, A. Drastic Changes in the Fluorescence Properties of NBD Probes with the Polarity of the Medium: Involvement of a TICT State? *J. Photochem. Photobiol. A Chem.* **1993**, *70*, 229–243.
